# Integrating machine learning and traditional statistics to evaluate the Uric acid-to-HDL cholesterol ratio for identification of prevalent COPD in older adults

**DOI:** 10.1186/s12944-026-03102-1

**Published:** 2026-10-27

**Authors:** Li Li, Chengfeng Fu, Ruqi Zhang, Minyan Zhang, Xinrui Tian

**Affiliations:** 1 Department of Geriatrics, The Second Hospital of Shanxi Medical University, Taiyuan, 030001 China; 2 The Second Affiliated Hospital, Chongqing Medical University, Chongqing, 400010 China

**Keywords:** Uric acid-to-HDL cholesterol ratio, Chronic obstructive pulmonary disease, NHANES, Older adults, Cross-sectional study

## Abstract

**Background:**

The uric acid-to-high-density lipoprotein cholesterol ratio (UHR) is a novel inflammatory and metabolic biomarker, but its association with chronic obstructive pulmonary disease (COPD) in older adults remains unclear. This study investigated the relationship between UHR and COPD prevalence among adults aged ≥ 60 years.

**Methods:**

This cross-sectional study analyzed 5,277 participants aged ≥ 60 years from NHANES 2013–2018. Weighted multivariable logistic regression estimated the UHR–COPD association. Restricted cubic spline (RCS) analysis characterized the dose–response relationship, and receiver operating characteristic (ROC) curves evaluated model performance. Subgroup, sensitivity, and machine learning analyses were conducted. Variables with missingness of 20% or higher were not imputed and were instead examined in complete-case analyses.

**Results:**

Of 5,277 participants, 404 (7.7%) had COPD. Elevated UHR was independently associated with higher COPD prevalence. In the fully adjusted model, each standard deviation (SD) increase in UHR corresponded to 23% higher odds (OR 1.23; 95% CI 1.05–1.44; *P* = 0.015). The highest versus lowest UHR quartile showed significantly higher risk (OR 1.71; 95% CI 1.09–2.68; P for trend = 0.019). RCS confirmed a linear dose–response relationship (P for non-linearity = 0.449). The model had good discrimination (AUC = 0.785). Adding UHR to 15 conventional predictors gave a small but statistically detectable increment in discrimination (AUC 0.784 to 0.788; DeLong *P* = 0.049), although the reclassification gains were small. After further adjustment for alcohol, the top-quartile association (OR 2.201, 95% CI 1.202 to 4.031; *P* = 0.018) and the quartile trend (*P* = 0.012) were retained, whereas the per-standard-deviation estimate was attenuated (OR 1.241, 95% CI 0.980 to 1.571; *P* = 0.085).

**Conclusions:**

Elevated UHR is associated with a higher prevalence of COPD in older adults. UHR may serve as a low-cost, routinely measurable marker to aid in the identification of prevalent, physician-diagnosed COPD in geriatric care. Because COPD status was ascertained at a single survey time point in a cross-sectional analysis, no prospective-screening inference is intended.

**Supplementary Information:**

The online version contains supplementary material available at https://doi.org/10.1186/s12944-026-03102-1.

## Introduction

Chronic obstructive pulmonary disease (COPD) is a common, preventable, and treatable respiratory disorder characterized by persistent airflow limitation and progressive symptoms [1]. It ranks among the leading causes of morbidity and mortality worldwide, with the greatest burden borne by older adults [2]. Population aging and the high prevalence of comorbid cardiometabolic disease, conditions that share inflammatory and oxidative-stress pathways with COPD [3] further add to this burden.

With aging, the lungs lose elastic recoil, mucociliary clearance weakens, and respiratory muscle strength slowly declines, leaving older adults more vulnerable to environmental insults and more susceptible to disease progression [4, 5]. A growing body of evidence links metabolic disturbances—including hyperuricemia, dyslipidemia, and abnormal glucose metabolism [6]—to both the development and course of COPD. These metabolic shifts appear to exacerbate lung injury by driving systemic oxidative stress [7] and inflammatory signaling [8], thereby connecting metabolic dysregulation to respiratory disease.

Serum uric acid (UA) [9, 10] and high-density lipoprotein cholesterol (HDL-C) [11] sit on opposite sides of redox balance and inflammation. At normal levels, UA acts as an antioxidant, but as levels rise it becomes pro-oxidant, largely through reactive oxygen species generated by xanthine oxidase [12]. HDL-C, in contrast, reduces inflammation and oxidative damage and facilitates cholesterol efflux from tissues. Combining the two into a ratio (UHR) captures both tendencies in a single value. UHR has already proved useful in predicting metabolic syndrome, diabetes, and cardiovascular disease [13], but whether it tracks COPD, particularly in older adults, remains largely unexplored [12, 14].

We therefore used NHANES 2013–2018, a nationally representative survey of U.S. adults aged 60 and older, to examine whether UHR is associated with COPD prevalence. We assessed the dose–response relationship, evaluated whether UHR adds discriminative value beyond standard predictors, and tested whether age, sex, smoking, or other factors modify the association. We hypothesized that higher UHR would be associated with higher COPD prevalence and would add information beyond conventional factors, consistent with metabolic redox imbalance contributing to chronic airway disease.

## Methods

### Study design and population

**Fig. 1:**
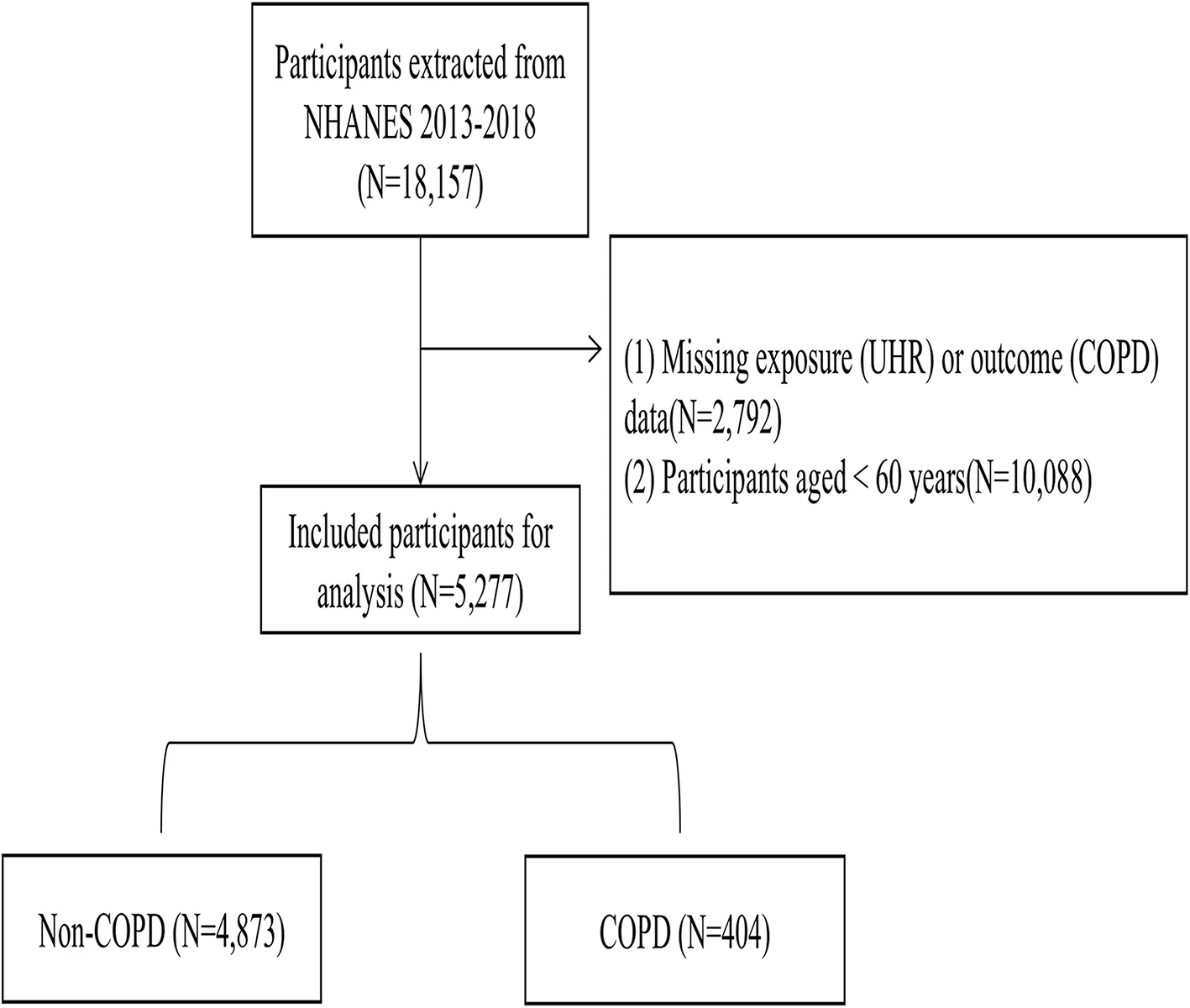
Flowchart of participant selection from NHANES 2013–2018. The source population comprised 18,157 adults after merging the DEMO, MCQ, BIOPRO, HDL, BMX, SMQ, DIQ, BPQ, CBC, and GHB datasets (6,266 in 2013–2014, 6,063 in 2015–2016, and 5,828 in 2017–2018). Participants with missing exposure (UHR) or outcome (COPD) data (*n* = 2,792) were excluded, leaving 15,365 participants, of whom 10,088 were younger than 60 years. The final analytic sample therefore comprised 5,277 adults aged 60 years or older, including 4,873 non-COPD participants and 404 participants with COPD

Data for this study were obtained from the NHANES from 2013 to 2018 (https://www.cdc.gov/nchs/nhanes/index.htm). NHANES interviewed 29,400 participants in the three survey cycles (10,175 in 2013–2014, 9,971 in 2015–2016, and 9,254 in 2017–2018). Of these, 28,242 completed the Sample Adult questionnaire containing the Medical Conditions module (9,770, 9,575, and 8,897 per cycle). Merging the 10 NHANES data modules (DEMO, MCQ, BIOPRO, HDL, BMX, SMQ, DIQ, BPQ, CBC, and GHB) by SEQN and retaining participants present in all modules left a pool of 18,157 (6,266, 6,063, and 5,828 per cycle). In the 2013–2014 cycle, after dropping 943 individuals with missing uric acid, HDL cholesterol, or self-reported COPD status, 5,323 remained; of these, 1,693 were aged 60 years or older and entered the analysis. The 2015–2016 cycle started with 6,063 participants. Missing exposure or outcome data led to the exclusion of 923 individuals, leaving 5,140, and restricting to adults aged 60 years or above gave 1,702. For 2017–2018, 5,828 participants were originally available; 926 were excluded because UHR or COPD data were missing, yielding 4,902, and 1,882 of them were aged 60 or older. Across the three cycles, 5,277 older adults were included, of whom 404 reported COPD. This study employed a cross-sectional design to evaluate the association between the serum uric acid to high-density lipoprotein cholesterol ratio (UHR) and COPD, along with other chronic lung diseases, in older adults. NHANES is a nationally representative health survey conducted by the National Center for Health Statistics (NCHS) under the U.S. Centers for Disease Control and Prevention (CDC). The survey utilizes a complex, multistage probability sampling design to ensure representativeness of the U.S. population. All participants provided written informed consent. Inclusion criteria were age 60 years or older, a valid response to the physician-diagnosed COPD questionnaire item (MCQ160O), and available serum uric acid and HDL-C measurements. Spirometry was neither required nor available: lung-function testing was last collected in NHANES in 2011–2012 and was not repeated in the 2013–2018 cycles. The 60-year threshold follows the World Health Organization definition of older adults [15] and matches the geriatric clinical setting in which the study was conceived. Exclusion criteria consisted of age under 60 years or missing UHR or COPD-related data. The participant selection flowchart is shown in Fig. 1. We selected the 2013–2018 cycles because they are the most recent NHANES waves that include all variables required for this study: fasting triglycerides, direct HDL cholesterol, serum uric acid, complete blood count, and the physician-diagnosed COPD questionnaire item (MCQ160O).

### Exposure variable

Serum UA and HDL-C were assayed from venous blood samples collected at the NHANES Mobile Examination Center (MEC). Serum samples were processed on-site and stored at − 30 °C until shipment to the CDC National Center for Environmental Health for analysis. UA was measured using a timed-endpoint enzymatic method on the Beckman Coulter UniCel DxC800 Synchron analyzer (uricase oxidation to allantoin and H₂O₂, followed by peroxidase-catalyzed color development read at 520 nm). HDL-C was determined by a direct enzymatic assay on the same platform, in which magnesium/dextran sulfate precipitation removed non-HDL fractions before PEG-modified cholesterol esterase and oxidase generated a chromogenic product measured at 600 nm [16, 17]. All analyses followed CDC quality control standards based on Westgard rules.

### Outcome variable

COPD was primarily defined based on NHANES questionnaire data. In the medical conditions section, participants were asked whether a doctor had ever told them they had COPD (variable MCQ160O). Individuals answering “yes” were classified as COPD cases, and those answering “no” as controls. Those who answered “Don’t know” (code 9) were excluded from the analysis [18, 19]. COPD status was based on self-reported physician diagnosis rather than spirometry. Lung function testing was not part of the NHANES 2013–2018 examination, and such self-reports have been shown to correspond well with clinically confirmed COPD in U.S. adults [20]; the potential for misclassification is acknowledged in the Discussion, together with the other limitations of the study. Older adults were defined according to the World Health Organization standard as individuals aged 60 years or older [15].

### Covariates

Covariates were selected based on literature review and biological relevance, including demographic characteristics, lifestyle factors, laboratory measurements, and comorbidities. Specific variables included age, sex, education level, family income-to-poverty ratio (PIR), body mass index (BMI), smoking status, alcohol use, triglycerides, hematocrit, platelet count, glycated hemoglobin (HbA1c), hypertension, coronary heart disease, diabetes, stroke, and any liver disease.

### Machine learning analysis

Participants were randomly divided into training (70%) and validation (30%) sets, and categorical variables were one-hot encoded. Six machine learning models, including Random Forest (RF), Extreme Gradient Boosting (XGBoost), Support Vector Machine (SVM), Gradient Boosting Decision Tree (GBDT), Logistic Regression (LR), and K-Nearest Neighbors (KNN), were constructed to predict COPD in older adults [21]. The optimal model was further analyzed using SHapley Additive exPlanations (SHAP) to quantify the contribution of each feature to model predictions and identify important predictive features.

### Statistical analysis

Missing data were handled using a pre-specified strategy. Variables with missing rates below 20% were imputed by multiple imputation. Variables with missing rates of 20% or higher were not imputed but were retained for the affected analyses, which were repeated as complete-case models. The 20% threshold is a safeguard on the validity of multiple imputation rather than an arbitrary filtering rule: multiple imputation assumes that data are missing at random and that the imputation model is correctly specified, and at high missingness the imputed values increasingly represent extrapolation, which can inflate uncertainty and bias [22, 23]. Descriptive statistics were presented as counts and percentages for categorical variables, and as mean ± standard error or median (interquartile range) for continuous variables depending on the distribution. Group differences were assessed using weighted χ² tests or weighted regression analyses. Weighted multivariable logistic regression models were applied to evaluate the association between UHR and COPD risk. Four nested models were constructed: Model 1 was unadjusted; Model 2 was adjusted for demographic and socioeconomic variables, including age, sex, education level, and family income-to-poverty ratio (PIR); Model 3 was further adjusted for lifestyle factors and comorbidities, including body mass index (BMI), smoking status, triglycerides, hematocrit, platelet count, glycated hemoglobin (HbA1c), hypertension, coronary heart disease, diabetes, stroke, and liver disease. Model 4 was further adjusted for alcohol use (ALQ130). In the present cohort the two variables exceeding this threshold did so for survey-design reasons: fasting triglycerides, which are available only in the morning fasting subsample, and the alcohol item ALQ130, which is administered only to participants reporting past-year drinking. Neither was imputed; triglycerides were addressed in the complete-case models (Table 3) and ALQ130 in the alcohol-adjusted Model 4 (Tables 2 and 4, and 5), and the primary and complete-case analyses were concordant. Prevalence odds ratios were estimated with the NHANES complex survey weights (WTINT2YR), incorporating the primary sampling-unit and strata variables, with nest set to TRUE.

UHR was incorporated as both a continuous variable (Z-score standardized) and a quartile-based categorical variable, with trend tests conducted. Restricted cubic spline (RCS) analysis was performed to assess the dose-response relationship between UHR and COPD. Model discrimination was evaluated using receiver operating characteristic (ROC) curves [24, 25]. To quantify the incremental value of UHR beyond conventional predictors, a base model comprising the 15 conventional covariates was compared with an extended model that additionally entered UHR per standard deviation, following the prediction-model evaluation framework applied to comparable indices in older-adult cohorts [26]. Because the improvement-index module used for this comparison does not accommodate survey weights, both models were fitted by unweighted maximum-likelihood estimation on the same analytic sample; discrimination was compared with the DeLong test, and the models were additionally compared by continuous and categorical net reclassification improvement and integrated discrimination improvement, estimated from 1,000 bootstrap resamples. Subgroup and sensitivity analyses were conducted to assess the robustness of the findings [27, 28]. In the machine learning analyses, models were trained and validated to evaluate predictive performance, and SHAP was used to interpret feature contributions. All analyses were performed using R (version 4.3.1) with the survey package and the Free Statistics software (versions 2.5 and 2.6.0; Beijing FreeClinical Medical Technology Co., Ltd, Beijing, China), and two-sided *P* < 0.05 was considered statistically significant.

## Results

### Baseline characteristics of study participants

A total of 5,277 participants aged ≥ 60 years were included in this study, of whom 404 (7.7%) had COPD and 4,873 (92.3%) served as non-COPD controls.

COPD participants had lower education and PIR [median 1.66 (IQR 1.04–2.65) vs. 2.16 (1.27–3.74)] and were more likely to be current or former smokers (85.6% vs. 46.7%; all *P* < 0.001; Table 1). BMI distributions did not differ (*P* = 0.315). Comorbidities were more frequent in the COPD group, most notably coronary heart disease (27.2% vs. 9.0%) and diabetes (36.9% vs. 27.4%; *P* < 0.001 for both). Hematocrit was marginally higher in COPD (*P* = 0.011); other lab parameters did not differ. The median UHR was higher in COPD [0.12 (0.08–0.16) vs. 0.11 (0.08–0.14), *P* < 0.001], with 36.9% of COPD cases in Q4 versus 24.0% of controls (*P* < 0.001).

**Table 1:** Baseline characteristics of participants in NHANES 2013–2018

Variable	Overall (*N* = 5277)	Non-COPD (*N* = 4873)	COPD (*N* = 404)	*P* value
**Age Group (years)**				0.003
60–75	3823 (72.4%)	3556 (73.0%)	267 (66.1%)	
> 75	1454 (27.6%)	1317 (27.0%)	137 (33.9%)	
**Gender**				< 0.001
Female	2673 (50.7%)	2512 (51.5%)	161 (39.9%)	
Male	2604 (49.3%)	2361 (48.5%)	243 (60.1%)	
**Education**				< 0.001
Less Than 9th Grade	747 (14.2%)	709 (14.5%)	38 (9.4%)	
9-11th Grade	667 (12.6%)	594 (12.2%)	73 (18.1%)	
High School Grad/GED or Equivalent	1247 (23.6%)	1122 (23.0%)	125 (30.9%)	
Some Colleges or AA degree	1463 (27.7%)	1342 (27.5%)	121 (30.0%)	
College Graduate or above	1153 (21.8%)	1106 (22.7%)	47 (11.6%)	
**PIR**	2.10 (1.25, 3.65)	2.16 (1.27, 3.74)	1.66 (1.04, 2.65)	< 0.001
**BMI**				0.315
Normal weight	1256 (23.8%)	1163 (23.9%)	93 (23.0%)	
Obese	2079 (39.4%)	1906 (39.1%)	173 (42.8%)	
Overweight	1942 (36.8%)	1804 (37.0%)	138 (34.2%)	
**Smoking**				< 0.001
No	2657 (50.4%)	2599 (53.3%)	58 (14.4%)	
Yes	2620 (49.6%)	2274 (46.7%)	346 (85.6%)	
**Hypertension**				0.007
No	2004 (38.0%)	1877 (38.5%)	127 (31.4%)	
Yes	3273 (62.0%)	2996 (61.5%)	277 (68.6%)	
**Diabetes**				< 0.001
No	3794 (71.9%)	3539 (72.6%)	255 (63.1%)	
Yes	1483 (28.1%)	1334 (27.4%)	149 (36.9%)	
**Coronary heart disease**				< 0.001
No	4728 (89.6%)	4434 (91.0%)	294 (72.8%)	
Yes	549 (10.4%)	439 (9.0%)	110 (27.2%)	
**Stroke**				0.002
No	4838 (91.7%)	4485 (92.0%)	353 (87.4%)	
Yes	439 (8.3%)	388 (8.0%)	51 (12.6%)	
**Liver disease**				0.016
No	4933 (93.5%)	4567 (93.7%)	366 (90.6%)	
Yes	344 (6.5%)	306 (6.3%)	38 (9.4%)	
**Hematocrit (%)**	41.20 (38.60, 43.70)	41.20 (38.60, 43.70)	41.80 (38.20, 44.90)	0.011
Mean platelet volume (fL)	8.30 (7.70, 8.90)	8.30 (7.70, 8.90)	8.20 (7.70, 8.80)	0.188
Glycohemoglobin (%)	5.80 (5.50, 6.30)	5.80 (5.50, 6.30)	5.90 (5.57, 6.50)	0.058
Triglycerides (mg/dL)	127.00 (90.00, 186.00)	127.00 (90.00, 186.00)	127.00 (91.00, 188.25)	0.696
**UHR**	0.11 (0.08, 0.14)	0.11 (0.08, 0.14)	0.12 (0.08, 0.16)	< 0.001
**UHR_quartile**				< 0.001
Q1 (0.02–0.08]	1320 (25.0%)	1239 (25.4%)	81 (20.0%)	
Q2 (0.08–0.11]	1319 (25.0%)	1234 (25.3%)	85 (21.0%)	
Q3 (0.11–0.14]	1320 (25.0%)	1231 (25.3%)	89 (22.0%)	
Q4 (0.14–0.56]	1318 (25.0%)	1169 (24.0%)	149 (36.9%)	

Note: Continuous variables were expressed as medians with interquartile ranges, whereas categorical variables were described as counts and percentages. Differences between the COPD and non-COPD groups were assessed using the chi-square test for categorical variables and the Mann-Whitney U test for continuous variables. The diagnosis of COPD was ascertained through a self-reported physician diagnosis of COPD (variable MCQ160O, the NHANES medical-conditions item on physician-diagnosed COPD, which is distinct from the chronic bronchitis item MCQ160K); those answering “Don’t know” (code 9) were excluded. UHR represents the ratio of serum uric acid concentration to HDL cholesterol level, which has been proposed as a composite marker reflecting oxidative stress and metabolic imbalance

Abbreviations: NHANES, National Health and Nutrition Examination Survey; COPD, Chronic Obstructive Pulmonary Disease; PIR, Poverty-Income Ratio; BMI, Body Mass Index; GED, General Educational Development; AA, Associate of Arts; UHR, Uric Acid-to-High-Density Lipoprotein Cholesterol Ratio; HDL, High-Density Lipoprotein; IQR, Interquartile Range; Q1, First Quartile; Q2, Second Quartile; Q3, Third Quartile; Q4, Fourth Quartile

### Association between UHR and COPD

Prior to constructing multivariable models, multicollinearity among all covariates was assessed using the variance inflation factor (VIF). All VIF values were well below 5, indicating no significant multicollinearity (see Additional file 1). Weighted logistic regression analyses showed that when UHR was treated as a continuous variable (Z-score standardized), the unadjusted model (Model 1) indicated that each 1-SD increase in UHR was associated with a 32% higher odds of COPD (OR 1.32, 95% CI 1.17–1.50, *P* < 0.001). In Model 2, adjusted for demographic factors, the association remained significant (OR 1.23, 95% CI 1.07–1.41, *P* = 0.005). In the fully adjusted model (Model 3), further controlling for smoking, comorbidities, and laboratory parameters, higher UHR remained positively associated with COPD prevalence (OR 1.23, 95% CI 1.05–1.44, *P* = 0.015).

When UHR was analyzed as quartiles, using Q1 as the reference, participants in Q4 had a significantly increased odds of COPD in the fully adjusted model (OR 1.71, 95% CI 1.09–2.68, *P* = 0.030), with a significant dose–response trend (*P* for trend = 0.019), indicating a positive linear association (Table 2). To further address confounding by alcohol consumption, Model 3 was additionally adjusted for alcohol use (ALQ130), yielding Model 4. This analysis included 2,717 complete cases (ALQ130 missing in 48.5%); the highest UHR quartile remained significantly associated with COPD (OR 2.20, 95% CI 1.20–4.03, *P* = 0.018), confirming that the UHR–COPD association is robust to adjustment for alcohol use (Table 2).

**Table 2:** Association analysis between UHR and COPD prevalence among older adults

	Model 1 OR (95% CI)	*P* value	Model 2 OR (95% CI)	*P* value	Model 3 OR (95% CI)	*P* value	Model 4 OR (95% CI)	Model 4 *P* value
**Continuous UHR Quartiles**	1.32 ( 1.17–1.50 )	< 0.001	1.23 ( 1.07–1.41 )	0.005	1.23 ( 1.05–1.44 )	0.015	1.241 ( 0.980–1.571 )	0.085
Q1	Reference		Reference		Reference		Reference	
Q2	0.96 (0.62–1.49)	0.860	0.84 (0.55–1.29)	0.441	0.84 (0.54–1.32)	0.464	0.826 ( 0.486–1.404 )	0.487
Q3	1.26 (0.81–1.96)	0.307	1.11 (0.71–1.74)	0.656	1.22 (0.70–2.10)	0.487	1.283 ( 0.625–2.636 )	0.504
Q4	2.15 (1.44–3.23)	< 0.001	1.74 (1.15–2.62)	0.012	1.71 (1.09–2.68)	0.030	2.201 (1.202–4.031)	0.018
***P*** **for trend**		< 0.001		0.011		0.019		0.012

Note: Model 1 was unadjusted. Model 2 was adjusted for demographic and socioeconomic variables, including age, sex, education level, and family income-to-poverty ratio (PIR). Model 3 was further adjusted for lifestyle factors and comorbidities, including body mass index (BMI), smoking status, triglycerides, hematocrit, platelet count, glycated hemoglobin (HbA1c), hypertension, coronary heart disease, diabetes, stroke, and liver disease. Model 4 was further adjusted for alcohol use (ALQ130). Because ALQ130 was missing for 48.5% of participants, as it was asked only of past-year drinkers, this model included 2,717 complete cases

### RCS analysis

**Fig. 2:**
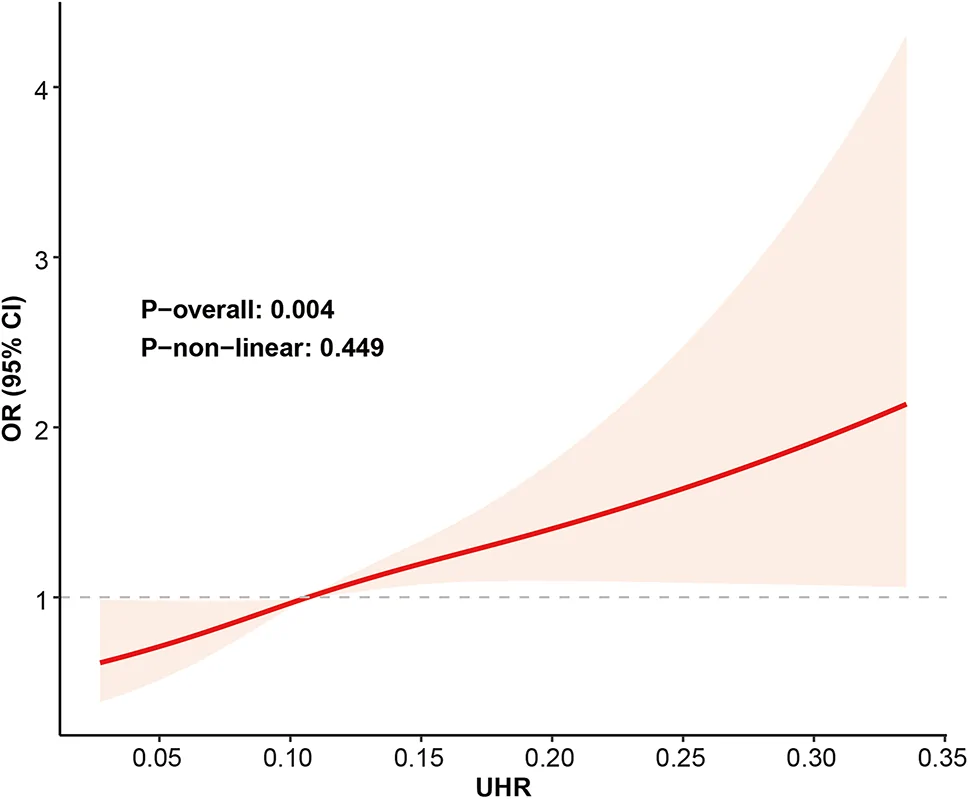
Association between UHR and COPD risk in older adults

RCS regression based on Model 3 was conducted to further assess the relationship between UHR and COPD prevalence. The results indicated a significant linear association, with no evidence of nonlinearity (*P*non-linear = 0.449), suggesting a linear dose–response relationship between UHR and COPD risk (Fig. 2).

### UHR distribution by COPD status

Boxplot analysis of UHR by COPD status showed that the median UHR was higher in the COPD group than in the non-COPD group, with an overall shift of the distribution toward higher values. The difference between groups was statistically significant (*P* < 0.001) (see Additional file 2).

### Subgroup analysis

**Fig. 3:**
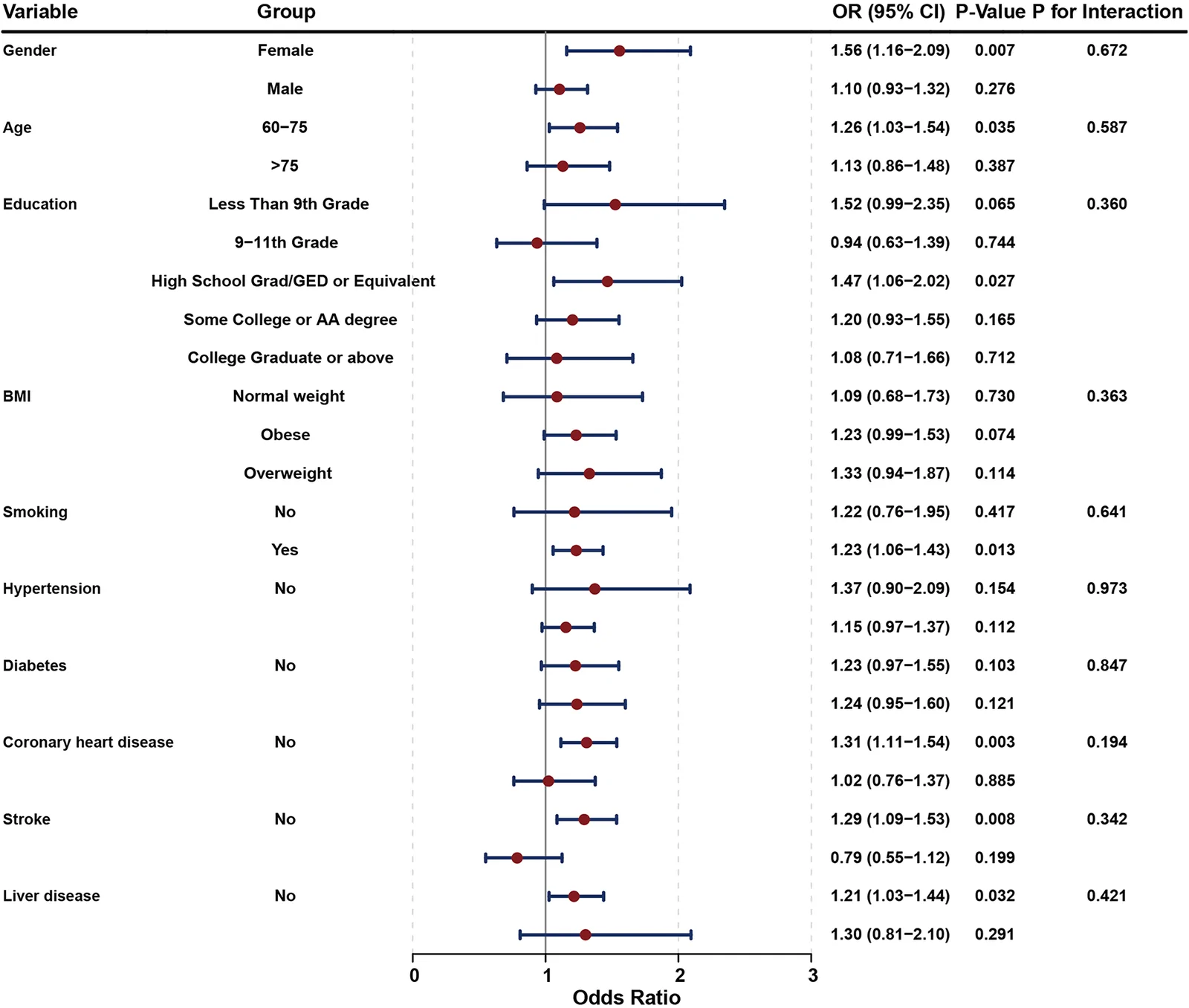
Subgroup analysis of the association between UHR and COPD risk in older adults. Forest plot showing ORs and 95% CIs across different subgroups (gender, age, education, BMI, smoking, hypertension, diabetes, coronary heart disease, stroke, liver disease). Interaction P values are shown. The vertical dashed line marks OR = 1

Subgroup analyses were conducted by 10 stratification variables: sex, age group, education level, BMI category, smoking status, hypertension, diabetes, coronary heart disease, stroke, and liver disease. The positive association between UHR and COPD risk was generally consistent across most strata. Significant associations were observed among females (OR 1.56, 95% CI 1.16–2.09), participants aged 60–75 years (OR 1.26, 95% CI 1.03–1.54), smokers (OR 1.23, 95% CI 1.06–1.43), and participants without a history of coronary heart disease or stroke. No significant interactions were detected for any stratification variables (*P* for interaction > 0.05), indicating that the association between UHR and COPD was robust across subgroups (Fig. 3).

### ROC analysis

**Fig. 4:**
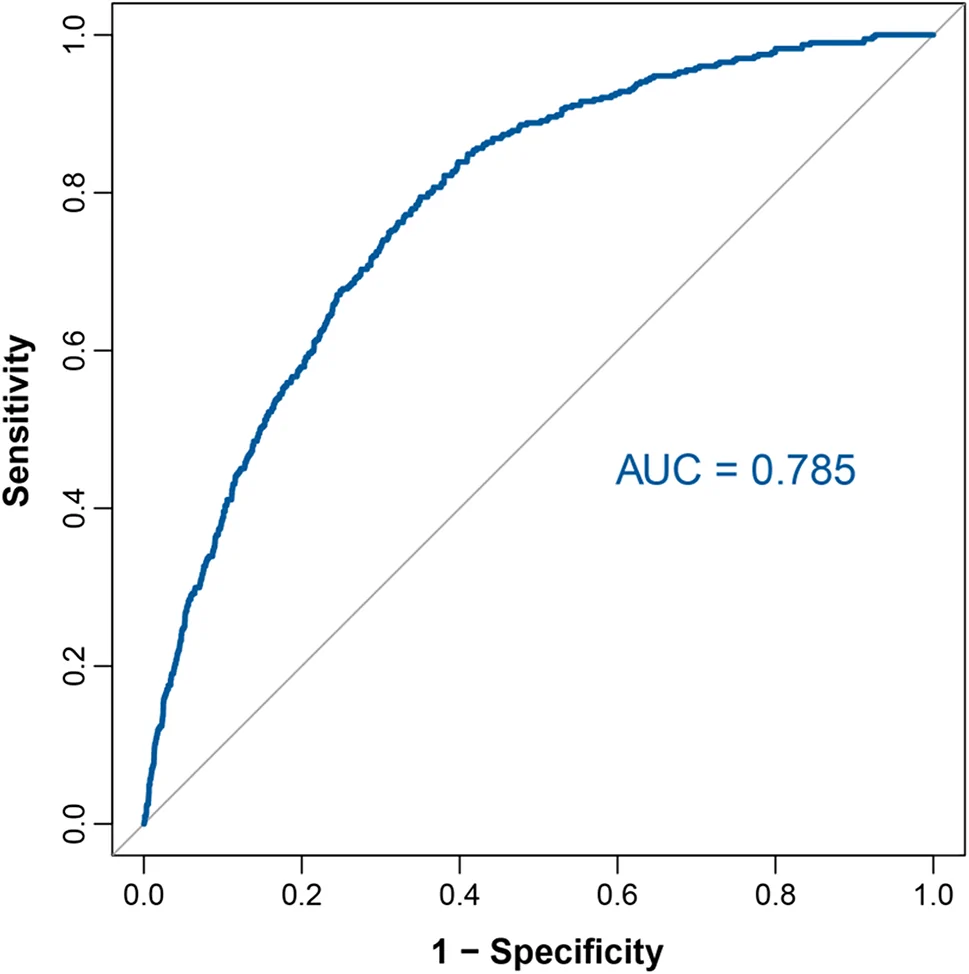
ROC curves for UHR in predicting COPD risk

The ROC curve for the fully adjusted logistic model (Model 3) yielded an AUC of 0.785 (Fig. 4), indicating that the multivariable model incorporating UHR, demographics, lifestyle factors, and comorbidities distinguishes COPD cases from controls with reasonable accuracy.

**Fig. 5:**
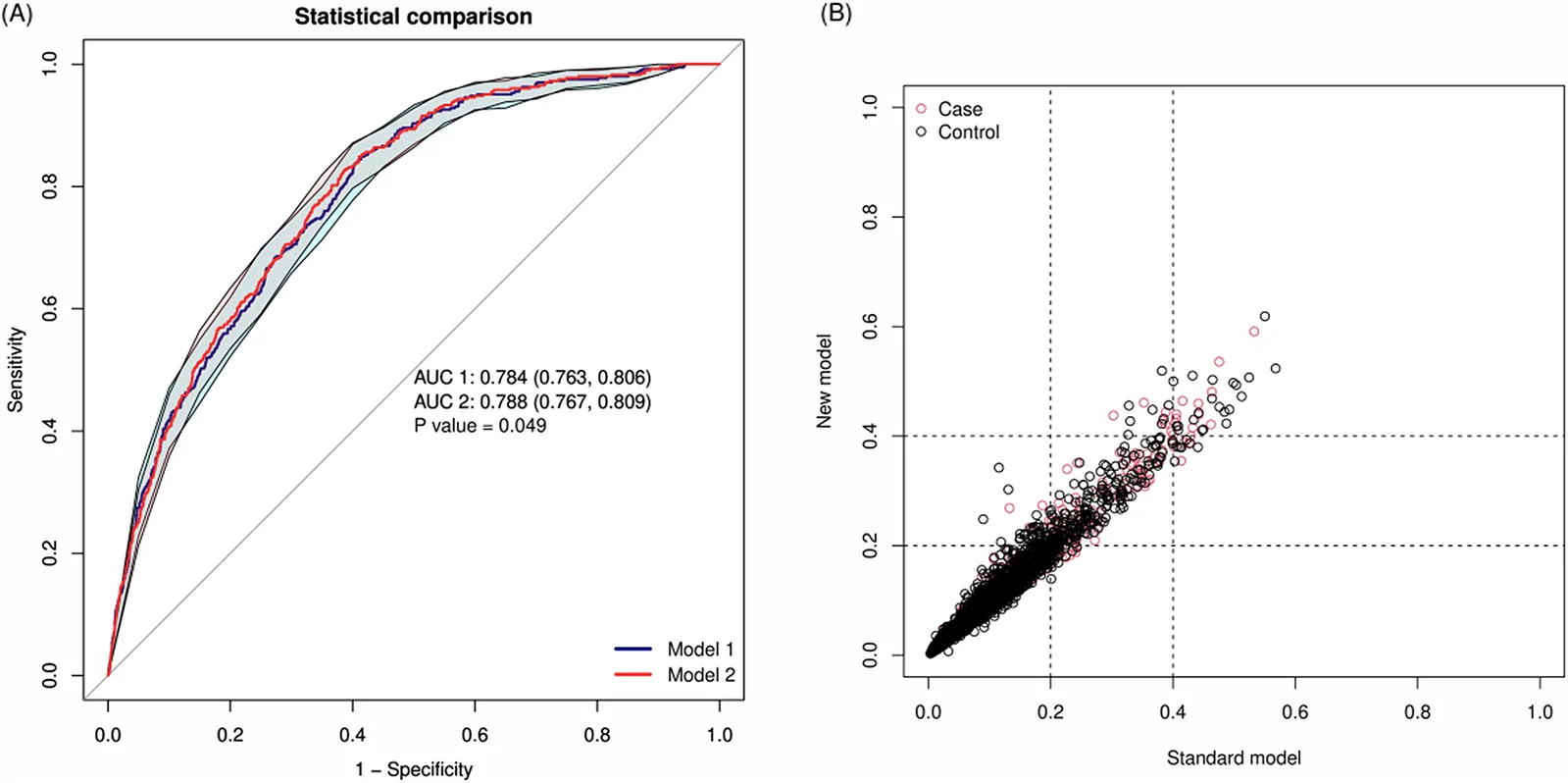
Discrimination and reclassification after adding UHR to the base model. (A) ROC curves for the base model (the 15 conventional covariates without UHR) and the extended model (the base model plus UHR per 1-SD increase) for predicting COPD among older U.S. adults, NHANES 2013–2018; shaded bands are 95% confidence intervals; AUC 0.784 (0.763–0.806) for the base model and 0.788 (0.767–0.809) for the extended model, DeLong *P* = 0.049. (B) Reclassification plot for the categorical NRI: predicted probability from the base model (x-axis) against the extended model (y-axis); dashed lines mark the 0.2 and 0.4 risk thresholds; red circles are cases and black circles are controls

We also examined whether adding UHR improved risk discrimination beyond the 15 conventional covariates. A logistic model including UHR (per 1-SD) achieved an AUC of 0.788 (95% CI 0.767 to 0.809), compared with 0.784 (95% CI 0.763 to 0.806) for the same covariates without UHR (ΔAUC 0.0038, DeLong *P* = 0.049; Fig. 5A). The improvement was smaller for the other indices: IDI 0.0023 (bootstrap 95% CI -0.0002 to 0.0047, *P* = 0.068) and continuous NRI 0.0962 (95% CI -0.0048 to 0.1972, *P* = 0.062), whereas the categorical NRI was essentially unchanged at -0.0043 (95% CI -0.0328 to 0.0242, *P* = 0.767; Fig. 5B). These estimates were obtained from unweighted model fits using the FreeStatistics ROC and improvement-index routines (1,000 bootstrap resamples for the indices; survey weights were applied only to the prevalence odds ratios reported in the tables) and indicate that UHR contributes a small but statistically detectable increment to discrimination beyond established predictors [26, 29, 30].

### Machine learning model construction and performance evaluation

**Fig. 6:**
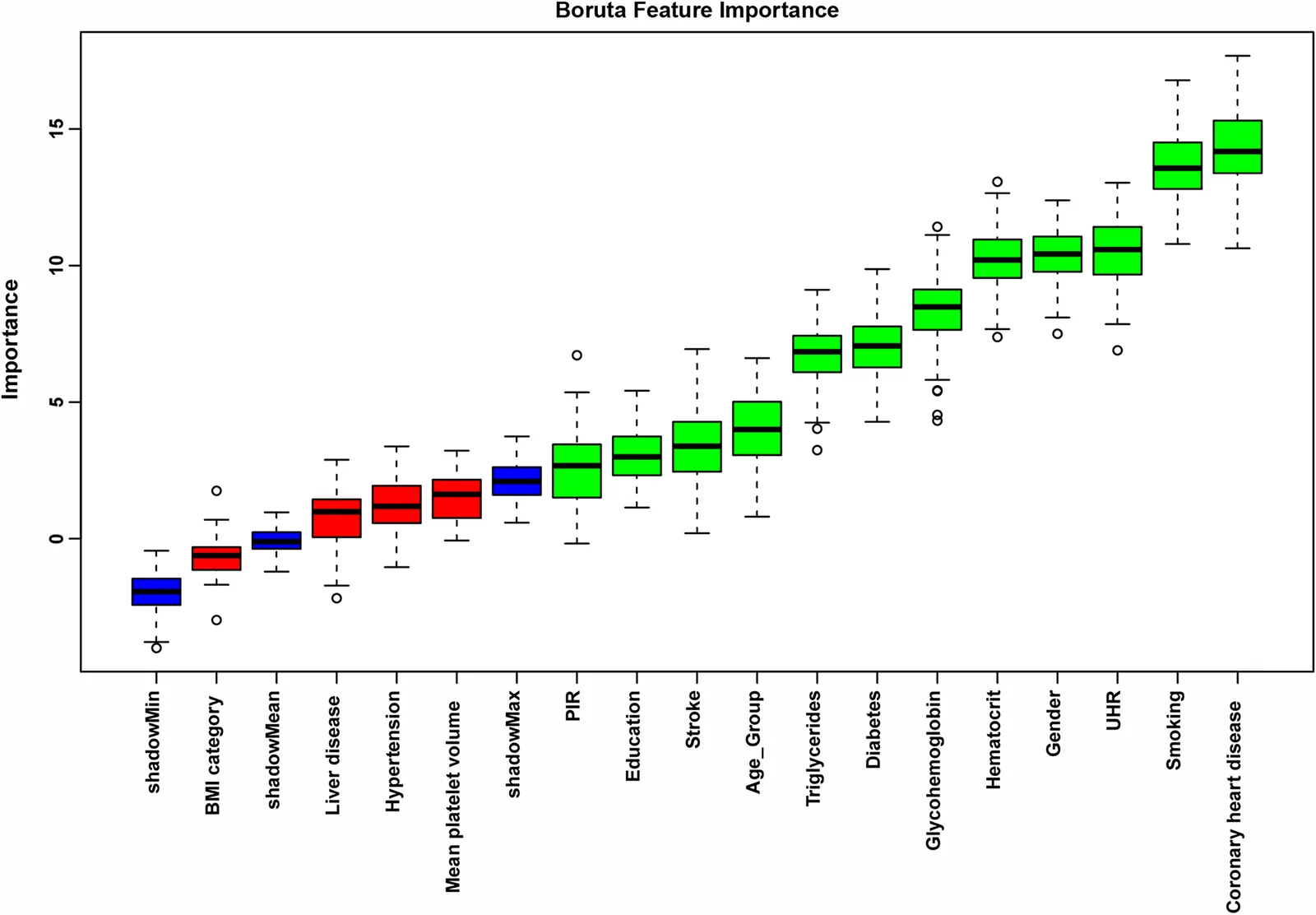
Feature selection based on the Boruta algorithm

Boruta feature selection identified 12 important variables with importance exceeding that of shadow variables (Fig. 6). The selected variables included UHR, smoking status, sex, age group, education level, PIR, hematocrit, HbA1c, triglycerides, diabetes, stroke, and coronary heart disease.

**Fig. 7:**
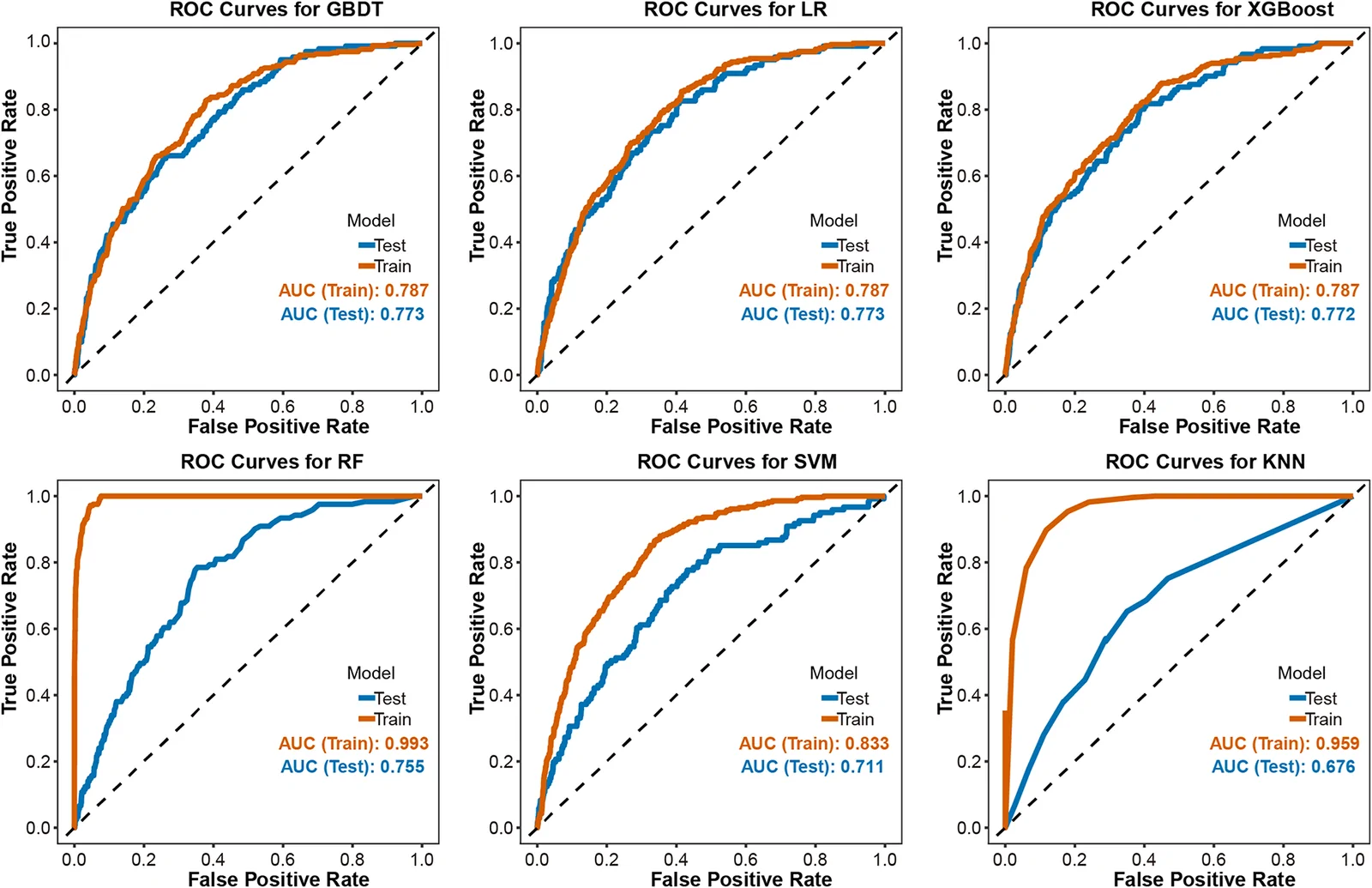
ROC curves of six machine learning models in training and validation sets

Six machine learning models, including RF, XGBoost, SVM, GBDT, LR, and KNN, were trained using the selected variables and evaluated in the validation set (Fig. 7, see Additional file 3). AUC values for GBDT, LR, and XGBoost were 0.7729, 0.7725, and 0.7719, respectively; AUCs for RF, SVM, and KNN were 0.7549, 0.7109, and 0.6756. In terms of classification performance, GBDT had an accuracy of 0.8692, sensitivity of 0.4215, and specificity of 0.9062; LR had an accuracy of 0.8622, sensitivity of 0.4215, and specificity of 0.8987.

Calibration curves (see Additional file 4) showed that RF and XGBoost remained closest to the ideal diagonal, whereas SVM, GBDT, and especially LR showed larger deviations, and all six models tended to over-predict COPD probability across the observed range. Decision curve analysis (see Additional file 5) indicated positive net benefit for all models at low threshold probabilities, with RF and XGBoost showing the highest net benefit and LR the lowest, and net benefit declining at higher threshold probabilities. Because discrimination was nearly identical across models (AUC 0.7719–0.7729) and calibration was not the basis for model selection, the LR model was selected for SHAP analysis on the basis of its interpretability.

#### SHAP analysis

**Fig. 8:**
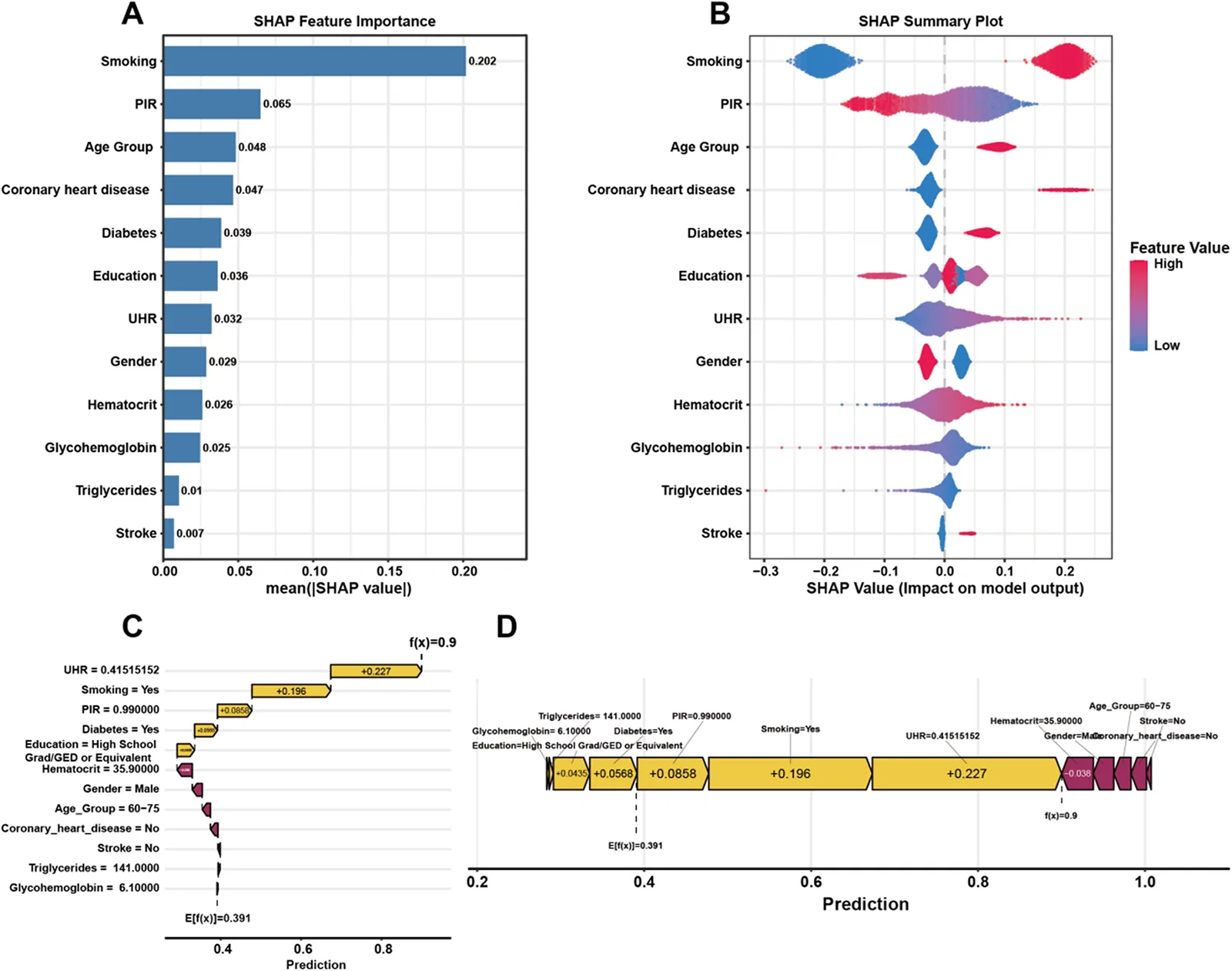
SHAP analysis for model interpretation. (**A**) Summary plot; (**B**) beeswarm plot; (**C**) waterfall plot; (**D**) force plot

SHAP analysis was conducted based on the LR model (Fig. 8). Global feature importance analysis (Fig. 8A) showed that the average contribution to model prediction varied across features, specifically smoking (mean |SHAP| = 0.202), PIR (0.065), age group (0.048), and UHR (0.032). These results indicate that smoking contributed substantially more to model predictions than UHR (mean |SHAP| 0.202 vs. 0.032), and UHR should be interpreted as providing incremental value on top of, rather than replacing, established risk factors such as smoking.

The SHAP summary beeswarm plot (Fig. 8B) demonstrated variability in the direction of feature effects on individual predictions. Higher UHR values were predominantly associated with positive SHAP values, while lower values were associated with negative SHAP values. A SHAP waterfall plot (Fig. 8C) for a representative individual showed a baseline predicted probability of 0.391 and a final predicted probability of 0.900. Contributions from UHR (0.415) and smoking were + 0.227 and + 0.196, respectively, with PIR (0.99) at + 0.086, diabetes at + 0.057, and hematocrit at − 0.038. The SHAP force plot (Fig. 8D) displayed positive and negative contributions of features for the individual, with UHR and smoking showing the largest positive contributions.

### Sensitivity analyses

Three sensitivity analyses were performed to assess the robustness of the association between UHR and COPD. In the complete-case dataset, standardized UHR remained significantly associated with COPD in the fully adjusted model (Model 3) (OR 1.26, 95% CI 1.06–1.49, *P* = 0.013). Quartile analysis showed increased risk in Q4 versus Q1 (OR 1.71, 95% CI 1.03–2.83, *P* = 0.047), with a significant trend (P for trend = 0.030) (Table 3). After further adjustment for alcohol use (Model 4), the per-SD estimate was attenuated (OR 1.24, 95% CI 0.98–1.57, *P* = 0.085), whereas the Q4-versus-Q1 association persisted (OR 2.20, 95% CI 1.20–4.03, *P* = 0.018; P for trend = 0.012), consistent with precision loss owing to the 48.5% missingness of alcohol intake.

After Winsorization of UHR, the standardized UHR remained significantly associated with COPD (OR 1.24, 95% CI 1.06–1.46, *P* = 0.014). Quartile analysis yielded consistent results, with Q4 risk higher than Q1 (OR 1.71, 95% CI 1.09–2.68, *P* = 0.030) and a significant trend (*P* for trend = 0.019) (Table 4). The alcohol-adjusted model (Model 4) yielded similar point estimates (per-SD OR 1.26, 95% CI 0.99–1.61, *P* = 0.071; Q4 OR 2.20, 95% CI 1.20–4.04, *P* = 0.018; P for trend = 0.012).

Excluding participants with asthma, standardized UHR remained significantly associated with COPD (OR 1.24, 95% CI 1.08–1.43, *P* = 0.005). Quartile analysis showed Q4 versus Q1 risk increased (OR 2.02, 95% CI 1.32–3.10, *P* = 0.003), with a significant trend (*P* for trend = 0.005) (Table 5). The alcohol-adjusted model (Model 4) confirmed the robustness of the association (per-SD OR 1.35, 95% CI 1.11–1.64, *P* = 0.006; Q4 OR 2.72, 95% CI 1.44–5.13, *P* = 0.005; P for trend = 0.003).

In a further sensitivity analysis, we examined an alternative case definition. When the outcome was broadened to include physician-diagnosed chronic bronchitis in addition to physician-diagnosed COPD (MCQ160K or MCQ160O), the fully adjusted association of standardized UHR with the outcome was substantially attenuated and no longer statistically significant (OR 1.06, 95% CI 0.89–1.27) compared with the primary definition (OR 1.32, 95% CI 1.03–1.70; see Additional file 6), indicating that the observed association was specific to physician-diagnosed COPD rather than to chronic airway disease in general.

**Table 3:** Logistic regression analysis of the association between UHR and COPD using non-imputed data

	Model 1 OR (95% CI)	*P* value	Model 2 OR (95% CI)	*P* value	Model 3 OR (95% CI)	*P* value	Model 4 OR (95% CI)	Model 4 *P* value
**Continuous UHR Quartiles**	1.37 (1.18–1.59)	< 0.001	1.28 (1.09–1.50)	0.004	1.26 (1.06–1.49)	0.013	1.241 (0.980–1.571)	0.085
Q1	Reference		Reference		Reference		Reference	
Q2	1.05 (0.65–1.69)	0.330	0.94 (0.58–1.50)	0.782	0.93 (0.56–1.56)	0.793	0.826 (0.485–1.404)	0.487
Q3	1.37 (0.82–2.30)	0.230	1.21 (0.72–2.03)	0.484	1.26 (0.68–2.34)	0.474	1.283 (0.625–2.636)	0.504
Q4	2.32 (1.44–3.74)	0.001	1.87 (1.16–3.04)	0.015	1.71 (1.03–2.83)	0.047	2.201 (1.202–4.031)	0.018
***P*** **for trend**		0.002		0.012		0.030		0.012

Note: Model 1 was unadjusted. Model 2 was adjusted for demographic and socioeconomic variables, including age, sex, education level, and family income-to-poverty ratio (PIR). Model 3 was further adjusted for lifestyle factors and comorbidities, including body mass index (BMI), smoking status, triglycerides, hematocrit, platelet count, glycated hemoglobin (HbA1c), hypertension, coronary heart disease, diabetes, stroke, and liver disease. Model 4 was further adjusted for alcohol use (ALQ130); because ALQ130 was missing for 48.5% of participants (asked only of past-year drinkers), this model rested on 2,717 complete cases

**Table 4:** Logistic regression analysis of the association between UHR and COPD after Winsorization

	Model 1 OR (95% CI)	*P* value	Model 2 OR (95% CI)	*P* value	Model 3 OR (95% CI)	*P* value	Model 4 OR (95% CI)	Model 4 *P* value
**Continuous UHR Quartiles**	1.33 (1.17–1.51)	< 0.001	1.24 (1.08–1.42)	0.004	1.24 (1.06–1.46)	0.014	1.262 (0.991–1.607)	0.071
Q1	Reference		Reference		Reference		Reference	
Q2	0.96 (0.62–1.49)	0.859	0.84 (0.55–1.30)	0.440	0.84 (0.54–1.32)	0.464	0.822 (0.483–1.398)	0.477
Q3	1.26 (0.81–1.96)	0.306	1.11 (0.71–1.74)	0.655	1.22 (0.71–2.11)	0.487	1.291 (0.629–2.652)	0.494
Q4	2.15 (1.44–3.23)	< 0.001	1.74 (1.15–2.62)	0.013	1.71 (1.09–2.68)	0.030	2.204 (1.203–4.038)	0.018
***P*** **for trend**		< 0.001		0.011		0.019		0.012

Note: Model 1 was unadjusted. Model 2 was adjusted for demographic and socioeconomic variables, including age, sex, education level, and family income-to-poverty ratio (PIR). Model 3 was further adjusted for lifestyle factors and comorbidities, including body mass index (BMI), smoking status, triglycerides, hematocrit, platelet count, glycated hemoglobin (HbA1c), hypertension, coronary heart disease, diabetes, stroke, and liver disease. Model 4 was further adjusted for alcohol use (ALQ130); because ALQ130 was missing for 48.5% of participants (asked only of past-year drinkers), this model rested on 2,717 complete cases in the Winsorized dataset

**Table 5:** Logistic regression analysis after excluding participants with asthma

	Model 1 OR (95% CI)	*P* value	Model 2 OR (95% CI)	*P* value	Model 3 OR (95% CI)	*P* value	Model 4 OR (95% CI)	Model 4 *P* value
**Continuous UHR Quartiles**	1.40 (1.26–1.55)	< 0.001	1.26 (1.13–1.41)	< 0.001	1.24 (1.08–1.43)	0.005	1.347 (1.109–1.636)	0.006
Q1	Reference		Reference		Reference		Reference	
Q2	1.32 (0.76–2.30)	0.332	1.12 (0.66–1.90)	0.679	1.14 (0.68–1.92)	0.622	1.028 (0.512–2.065)	0.940
Q3	1.70 (1.02–2.85)	0.049	1.38 (0.84–2.28)	0.211	1.47 (0.83–2.59)	0.197	1.141 (0.521-2.500)	0.744
Q4	2.90 (1.87–4.50)	< 0.001	2.13 (1.38–3.28)	0.002	2.02 (1.32–3.10)	0.003	2.718 (1.439–5.134)	0.005
***P*** **for trend**		< 0.001		0.004		0.005		0.003

Note: Model 1 was unadjusted. Model 2 was adjusted for demographic and socioeconomic variables, including age, sex, education level, and family income-to-poverty ratio (PIR). Model 3 was further adjusted for lifestyle factors and comorbidities, including body mass index (BMI), smoking status, triglycerides, hematocrit, platelet count, glycated hemoglobin (HbA1c), hypertension, coronary heart disease, diabetes, stroke, and liver disease. Model 4 was further adjusted for alcohol use (ALQ130); because ALQ130 was missing for 48.5% of participants (asked only of past-year drinkers), this model rested on 2,335 complete cases after excluding participants with asthma

## Discussion

In this cross-sectional analysis of adults aged 60 and older from NHANES 2013–2018, UHR rose steadily with COPD prevalence and the link was linear. The association held after adjusting for a wide set of potential confounders, and restricted cubic spline analysis confirmed the linear dose-response shape. Machine learning models also picked UHR out as a useful predictor of COPD in this older population.

The result fits the biology. Uric acid is an antioxidant at normal levels but turns pro-oxidant when it rises, largely by driving xanthine oxidase-mediated reactive oxygen species. HDL-C, by contrast, calms inflammation and oxidative damage in lung tissue [31]. UHR joins the two into one index, so it may capture the oxidant-antioxidant balance better than either marker on its own. Because oxidative stress and inflammation sit at the core of COPD, and because both worsen as lung recoil, mucociliary clearance, and respiratory muscle strength decline with age [14, 32], a higher UHR tracking with COPD is what the pathophysiology would predict. Our data build on work linking hyperuricemia [33], low HDL-C [34], and broader metabolic disturbance [35] to cardiometabolic disease by carrying UHR into chronic respiratory disease [36, 37]. That UHR did better than either single marker echoes metabolic and cardiovascular studies and adds to evidence that metabolic derangement helps drive COPD susceptibility.

Several recent studies point to a cardiometabolic-inflammatory basis for the UHR-COPD association. Bao et al. (2025) reported, in a nationally representative U.S. sample, that a higher uric acid-to-HDL ratio was linked to greater COPD risk [12] using the same ratio we examined. Rao et al. (2024) reached a similar conclusion for serum urate itself, both in a hospital cohort and by Mendelian randomization [38], and Zhang et al. (2024) found that urate together with a healthy diet predicted COPD incidence in the UK Biobank [32]. Taken together, these reports agree with our results: higher urate and altered HDL appear to mark a low-grade, pro-oxidant inflammatory state that raises the risk of COPD.

In our data the UHR-COPD link held in most subgroups, but the effect was stronger in women, in those aged 60–75, in current smokers, and in people without prior coronary heart disease or stroke. Other population studies have found similar patterns: composite uric-acid metrics track airflow limitation and COPD, and the size of the effect shifts with age, sex, and exposure.

Sex differences probably run through more than estrogen. After menopause, falling estrogen changes both HDL levels and how the kidney handles uric acid, which can make UHR a louder signal of oxidative stress and inflammation in women [12, 39]. Other studies have also found stronger ties between high uric acid or related ratios and airflow obstruction in women. So UHR may be picking up a sex-specific metabolic-inflammatory vulnerability that helps drive COPD.

Estrogen is unlikely to be the whole explanation, however. Androgens also affect uric acid handling and the composition of HDL particles, and the well-described sex difference in fat distribution changes the inflammatory and adipokine background against which UHR should be interpreted. Smoking exposure, which differs between men and women, adds another layer to the airway-inflammatory setting where UHR operates. Because HDL composition itself varies by sex, the same UHR value may not carry identical metabolic meaning in men and women.

The stronger link in the 60–75 group probably reflects the buildup of oxidative stress, low-grade inflammation, and metabolic disturbance that comes with age, all of which make the lung more vulnerable and may give UHR more prognostic weight in older adults [38, 40]. For smoking, tobacco directly inflames the airway and disrupts systemic metabolism, changing how uric acid is made and cleared and how HDL is composed, which would strengthen the UHR-COPD tie in current smokers. Large cohorts report that serum urate interacts with lifestyle factors such as smoking and diet in COPD, so behavioral exposures can amplify metabolically driven respiratory outcomes [32, 38].

No stratification variable showed a significant interaction (P for interaction > 0.05): the effect size varies across subgroups, but the UHR-COPD association holds throughout. That matches cohort and Mendelian randomization work finding a steady positive link between urate-related indices and COPD after heavy confounder adjustment [12, 38]. In practice, UHR looks like a broadly usable metabolic-inflammatory marker for spotting COPD, and it may work best in older adults, women, and current smokers.

Future work should test these subgroup effects in prospective cohorts and in mechanistic studies that show how uric acid and HDL-C meet smoking, sex hormones, and aging to shape airway inflammation. Trials that lower UHR through lifestyle change, urate-lowering drugs, or HDL-targeted therapy, and then track COPD progression, would also be informative. Sorting out the direct lung effects of UHR will require controlling for cardiovascular comorbidity and the drugs used to treat it [12, 38–40].

This study has several strengths. Its main strength is the nationally representative, survey-weighted sample, which supports generalization, together with full multivariable adjustment, RCS dose-response modeling, and machine-learning cross-checks on the robustness of the UHR finding.

This study has several limitations. First, the cross-sectional design cannot establish causality or temporal order. Second, COPD was defined by self-reported physician diagnosis (MCQ160O) rather than spirometry, so outcome misclassification is possible and comparability with spirometry-based case definitions is limited; because lung-function testing was last collected in NHANES in 2011–2012, spirometric confirmation was not possible in the 2013–2018 cycles. Third, UHR and its components were derived from a single blood draw, which may not reflect longer-term exposure, and urate- and lipid-lowering medication may have shifted the biomarkers despite statistical adjustment. Fourth, the alcohol item ALQ130 was administered only to participants reporting past-year drinking, so the alcohol-adjusted model rested on 2,717 complete cases and its precision was correspondingly lower; complete-case analyses for the two variables with missingness of 20% or higher may also be subject to selection bias. Fifth, the sample was drawn from a United States survey, which limits generalization to other ethnic and national settings. Finally, we did not measure C-reactive protein or any other inflammatory marker, so we could not test whether UHR reflects an inflammatory pathway independent of smoking, and COPD was not confirmed by lung function. Because COPD status was ascertained at a single survey time point, the findings concern prevalent, physician-diagnosed COPD and do not support prospective screening or risk stratification; longitudinal cohorts with spirometry and inflammatory biomarkers are needed.

Our findings suggest that UHR could serve as an inexpensive, readily available marker to aid in the identification of prevalent COPD in older adults. Because it integrates both pro-oxidant and antioxidant signals, UHR may better reflect the metabolic redox imbalance underlying chronic airway inflammation than any single metabolite. Translating this association into clinical application will require longitudinal cohorts with repeated measurements and a standardized spirometric definition of COPD [41, 42], as well as trials assessing whether reducing UHR through lifestyle changes or antioxidant therapy [43] improves respiratory outcomes. Mechanistic studies of xanthine oxidase activity and HDL function in lung remodeling would help clarify the underlying link.

## Conclusion

In adults aged ≥ 60 years, higher UHR was linearly associated with greater COPD prevalence, and this association remained significant after multivariable adjustment and in sensitivity analyses. UHR added a small but statistically detectable increment to discrimination when added to conventional predictors and should therefore be regarded as a complementary marker rather than a dominant risk factor. Since UHR can be derived from routine blood tests, it may aid in the identification of prevalent, physician-diagnosed COPD in older adults; because the data are cross-sectional and COPD status was recorded at a single survey time point, no prospective-screening inference is intended. Prospective studies are needed to confirm causality and establish clinically actionable thresholds.

## Supplementary Information

Below is the link to the electronic supplementary material.

Supplementary Material 1: Additional file 1. Variance inflation factors (VIF) for covariates in the multivariable logistic regression models. All VIF values were well below 5, indicating no significant multicollinearity among the covariates.

Supplementary Material 2: Additional file 2. Boxplot of the uric acid-to-high-density lipoprotein cholesterol ratio (UHR) by COPD status among older U.S. adults (NHANES 2013–2018).

Supplementary Material 3: Additional file 3. Performance of the six machine learning models (RF, XGBoost, SVM, GBDT, LR, and KNN) in the validation set, including AUC, accuracy, precision, sensitivity, specificity, and F1 score.

Supplementary Material 4: Additional file 4. Calibration curves of the six machine learning models in the validation set.

Supplementary Material 5: Additional file 5. Decision curve analysis of the six machine learning models in the validation set.

Supplementary Material 6: Additional file 6. Sensitivity analysis of the association between UHR and COPD using an alternative COPD case definition (physician-diagnosed COPD or chronic bronchitis), with survey weighting and full adjustment.

## Data Availability

The data used in this study are publicly available from the National Health and Nutrition Examination Survey (NHANES) at https://www.cdc.gov/nchs/nhanes/index.html.

## Abbreviations

UHR: Uric acid-to-high-density lipoprotein cholesterol ratio
COPD: Chronic obstructive pulmonary disease
NHANES: National Health and Nutrition Examination Survey
RCS: Restricted cubic spline
ROC: Receiver operating characteristic
SD: Standard deviation
HDL-C: High-density lipoprotein cholesterol
CDC: The U.S. Centers for Disease Control and Prevention
PIR: Poverty ratio
BMI: Body mass index
HbA1c: Glycated hemoglobin
RF: Random Forest
XGBoost: Extreme Gradient Boosting
SVM: Support Vector Machine
GBDT: Gradient Boosting Decision Tree
LR: Logistic Regression
KNN: K-Nearest Neighbors
SHAP: SHapley Additive exPlanations
AUC: An area under the curve
VIF: variance inflation factor
