# Supplementary material for: Integrating machine learning and traditional statistics to evaluate the Uric acid-to-HDL cholesterol ratio for identification of prevalent COPD in older adults
Source: Lipids Health Dis. 2026 Oct 27;25:221. doi: 10.1186/s12944-026-03102-1 (PMC13741772; doi:10.1186/s12944-026-03102-1)
Supplement: Supplementary file 1 [file 12944_2026_3102_MOESM1_ESM.pdf]

Additional file 1. Variance inflation factors (VIF) for covariates in the multivariable logistic regression models. All VIF values were well below 5, indicating no significant multicollinearity among the covariates.

Variable	VIF
Age_Group	1.04
Gender	1.18
Education	1.03
PIR	1.10
BMI_category	1.05
Smoking	1.03
Hypertension	1.05
Diabetes	1.29
Coronary_heart_disease	1.05
Stroke	1.02
Liver_disease	1.02
Hematocrit	1.11
Mean_platelet_volume	1.02
Triglycerides	1.11
Glycohemoglobin	1.26
UHR	1.21
