# Supplementary material for: Integrating machine learning and traditional statistics to evaluate the Uric acid-to-HDL cholesterol ratio for identification of prevalent COPD in older adults
Source: Lipids Health Dis. 2026 Oct 27;25:221. doi: 10.1186/s12944-026-03102-1 (PMC13741772; doi:10.1186/s12944-026-03102-1)
Supplement: Supplementary file 2 [file 12944_2026_3102_MOESM3_ESM.pdf]

Additional file 3. Performance of the six machine learning models (RF, XGBoost, SVM, GBDT, LR, and KNN) in the validation set, including AUC, accuracy, precision, sensitivity, specificity, and F1 score.

AUC, area under the receiver operating characteristic curve. Models are sorted by AUC. GBDT, gradient boosting decision tree; LR, logistic regression; KNN, K-nearest neighbors; RF, random forest; SVM, support vector machine; XGBoost, extreme gradient boosting.

Model	AUC	Accuracy	Precision	Sensitivity	Specificity	F1 score
**GBDT**	0.7729	0.8692	0.2713	0.4215	0.9062	0.3301
**LR**	0.7725	0.8622	0.2563	0.4215	0.8987	0.3188
**XGBoost**	0.7719	0.8609	0.2562	0.4298	0.8966	0.3210
**RF**	0.7549	0.8407	0.2063	0.3802	0.8789	0.2674
**SVM**	0.7109	0.8350	0.1957	0.3719	0.8734	0.2564
**KNN**	0.6756	0.7004	0.1405	0.5702	0.7112	0.2255
