# Supplementary material for: Integrating machine learning and traditional statistics to evaluate the Uric acid-to-HDL cholesterol ratio for identification of prevalent COPD in older adults
Source: Lipids Health Dis. 2026 Oct 27;25:221. doi: 10.1186/s12944-026-03102-1 (PMC13741772; doi:10.1186/s12944-026-03102-1)
Supplement: Supplementary file 3 [file 12944_2026_3102_MOESM6_ESM.pdf]

Additional file 6. Sensitivity analysis of the association between UHR and COPD using an alternative COPD case definition (physician-diagnosed COPD or chronic bronchitis), with survey weighting and full adjustment.

Model 3 was adjusted for age, sex, education level, PIR, BMI, smoking status, triglycerides, hematocrit, platelet count, HbA1c, and UA.

Case definition	n	COPD cases, n (%)	OR (95% CI)	P
Physician-diagnosed COPD (MCQ160O; primary definition)	2,042	168 (8.2)	1.323 (1.028-1.704)	0.031
Physician-diagnosed COPD or chronic bronchitis (MCQ160O or MCQ160K; broadened definition)	2,036	270 (13.3)	1.061 (0.889-1.266)	0.497
